# Characterization of ADAMTS13 and von Willebrand factor levels in septic and non-septic ICU patients

**DOI:** 10.1371/journal.pone.0247017

**Published:** 2021-02-19

**Authors:** Kanwal Singh, Andrew C. Kwong, Hasam Madarati, Sharumathy Kunasekaran, Taylor Sparring, Alison E. Fox-Robichaud, Patricia C. Liaw, Colin A. Kretz

**Affiliations:** 1 Department of Health Sciences, Thrombosis and Atherosclerosis Research Institute (TaARI), McMaster University, Hamilton, Ontario, Canada; 2 Department of Medicine, McMaster University, Hamilton, Ontario, Canada; Institut d’Investigacions Biomediques de Barcelona, SPAIN

## Abstract

Sepsis is a life-threatening disease characterized by excessive host response to infection that can lead to activation of the coagulation system. Von Willebrand Factor (VWF) and ADAMTS13 are important regulators of hemostasis and their dysregulation during sepsis progression is not well understood. Herein we characterize ADAMTS13 and VWF in septic and non-septic patients. ADAMTS13 activity, ADAMTS13 antigen, VWF antigen, myeloperoxidase, and protein C, were measured in plasma collected from 40 septic patients (20 non-survivors and 20 survivors) and 40 non-septic patients on the first and last day of their ICU stay. ADAMTS13 activity and ADAMTS13 antigen were reduced, whereas VWF antigen was elevated among septic patients compared to non-septic patients and healthy controls. Non-septic patients also exhibited elevated VWF antigen and reduced ADAMTS13 activity, but to a lesser extent than septic patients. Non-survivor septic patients exhibited the lowest levels of ADAMTS13 activity. ADAMTS13 activity:antigen ratio was similar across all patient cohorts suggesting that the specific activity of ADAMTS13 remains unchanged. Therefore, reduced ADAMTS13 function in circulation is likely due to a reduction in circulating levels. We suggest that massive release of VWF in response to inflammation consumes limited circulating ADAMTS13, resulting in the imbalance observed between VWF and ADAMTS13 among septic and to a lesser extent in non-septic ICU patients. Changes to ADAMTS13 did not correlate with myeloperoxidase or protein C levels. Reduced ADAMTS13 activity and antigen, and elevated VWF antigen observed among all patient cohorts on admission remained unchanged in survivors at ICU discharge. Prolonged reduction in ADAMTS13 activity and antigen in septic patients coincides with elevated levels of VWF. The persistent abnormalities in ADAMTS13 and VWF in sepsis patients discharged from the ICU may contribute to a sustained prothrombotic state.

## Introduction

Von Willebrand factor (VWF) is a large multimeric plasma protein that recruits platelets to sites of blood vessel injury [1]. VWF is secreted from endothelial cells and has a half-life of 15 hours [1, 2]. The platelet-binding capacity of VWF is proportional to the length of its multimer, with high molecular weight multimers possessing the greatest platelet-binding capacity and low molecular weight multimers the weakest platelet-binding [1]. Therefore, VWF multimer lengths must be tightly regulated in order to maintain a balanced hemostatic system. ADAMTS13 (*a d*isintegrin *a*nd *m*etalloprotease with *t*hrombo*s*pondin type 1 motifs, member *13*) is a circulating metalloprotease that that regulates the platelet binding capacity of VWF [1, 3]. ADAMTS13 is constitutively secreted as an active protease from hepatic stellate cells and has a circulating half-life of 3 days [3, 4]. Insufficient ADAMTS13 activity can lead to thrombotic thrombocytopenic purpura, a rare but devastating microvascular thrombotic disorder characterized by the spontaneous formation of VWF and platelet-rich microthrombi that form primarily in the kidney, gut, and brain [5].

Sepsis is defined as a life-threatening organ dysfunction caused by excessive host response to infection [6], and is a leading cause of mortality and critical illness worldwide [7]. Activation of blood coagulation during sepsis and septic shock can lead to disseminated intravascular coagulation (DIC), which is characterized by microvascular thrombosis, consumption of clotting factors and platelets, and bleeding [8]. Recent studies have suggested a reciprocal correlation between VWF and ADAMTS13 in sepsis [9]. The extent of reduced ADAMTS13 antigen levels and activity appeared strongly associated with sepsis severity and prognosis [9, 10]. The mechanism of ADAMTS13 dysregulation in sepsis is unknown, however it has been suggested to be a consequence of reduced synthesis [11], degradation by thrombin [12, 13], consumption by massive amounts VWF [14], or impaired proteolytic activity in the presence of inflammatory mediators [15, 16].

While improvements in ICU protocols have increased the likelihood of surviving sepsis, the lingering long-term effects of sepsis after discharge remain unclear. Many patients who survive sepsis have reduced quality of life and suffer from functional disability and cognitive impairment [17]. Longitudinal changes in the activity and antigen levels of ADAMTS13 and VWF throughout the course of sepsis still remain poorly characterized. Furthermore, distinguishing the levels of these proteins in sepsis from other ICU populations is not well described. The objective of this study is to characterize ADAMTS13 and VWF in septic and non-septic patients during the first and last day of ICU stay.

## Materials & methods

### Patients and inclusion criteria

Plasma samples from 40 septic ICU patients and 40 non-septic ICU patients were obtained from a prospective, multi-centre observational study of septic ICU patients (the DYNAMICS Study, Clinical Trials.gov Identifier: NCT01355042) [18]. The patients were recruited from ICUs in nine Canadian tertiary hospitals between November 2010 and January 2013. The study was approved by the HIREB (Hamilton Integrated Research Ethics Board); REB#: 10–532 (DYMANICS Study) and REB#: 12-712-T (obtaining blood from healthy volunteers). All patient samples were de-identified. All methods performed were in accordance with relevant guidelines/regulations, and written informed consent was obtained from the patient (or from the substitute decision maker if the patient was unable to provide consent). Consent form was witnessed and signed by the research coordinator. Patients were excluded if they were <18 years old, pregnant or breastfeeding, or were receiving palliative care. The inclusion criteria were a modification of those defined by Bernard et al. [19]. Patients must have a confirmed or suspected infection on the basis of clinical data at the time of screening, at least one dysfunctional organ system, ≥3 signs of systemic inflammatory response syndrome (SIRS), and were expected to remain in the ICU for ≥72 hours. The inclusion criteria for septic shock are the same as those for sepsis except that patients must be on vasopressors within the previous 24 hours. The inclusion criteria for non-septic patients were as follows: (a) expected to remain in the ICU for ≥72 hours and (b) were admitted with critical illness or had multiple traumas or had non-septic shock. Non-septic ICU patients were all survivors and were intended to be used for comparison alongside healthy individuals. Plasma samples were obtained from 10 healthy adult volunteers not receiving medication. No attempt to match cases and controls was made.

### Clinical data collection

Baseline characteristics include demographic information, organ function scores (MODS, SOFA), pre-existing conditions, sites and types of infection, APACHE II score, use of mechanical ventilation, and use of vasopressor/inotropes. Daily data included culture results, organ function, and hematologic and other laboratory tests.

### Blood collection

Patient blood samples were collected within 24 hours of meeting the inclusion criteria for sepsis or non-sepsis, then daily for the first week, followed by once a week for the duration of the patients’ ICU stay. Last day sample represents the last sample for the patient available (within last week period of patient discharge or death). The blood was processed into Becton Dickinson buffered sodium citrate vacutainer tubes (0.105M trisodium citrate). The blood was centrifuged at 1,700xg for 10 min at room temperature, and the plasma was stored in aliquots at -80°C.

### Measurement of parameters

ADAMTS13 activity was measured using FRETS-VWF73 (Anaspec Inc; Cat. #AS-63728-01; Fremont, USA). Total ADAMTS13 antigen in plasma was quantified using human ADAMTS13 Quantikine ELSIA kit (R&D Systems; Cat. #DADT130; Minneapolis, USA). VWF antigen was quantified using human VWF-A2 DuoSet ELISA (R&D Systems; Cat. #DY2764-05). Protein C antigen was quantified by ELISA (Affinity Biologicals Inc; Cat. #PC-EIA; Ancaster, Canada). MPO levels were quantified using human Myeloperoxidase Quantikine ELISA kit (R&D Systems; Cat. #DMYE00B). All commercial assays were conducted according to manufacturer’s protocols. ADAMTS13 activity, ADAMTS13 antigen, VWF antigen, PC antigen, and MPO values were expressed relative to healthy control plasma. All parameters were measured on Day 1 and Day Last (non-survivors septic: 10.6±8.8 days, survivors septic: 5.7±3.0 days, and non-septic: 5.0±2.2 days).

### Statistical analysis

Dunnett’s multiple comparisons test (one-way ANOVA) was performed to compare patient cohorts to healthy controls. Paired t-test was performed to assess biomarker change between Day 1 and Day Last. Pearson correlation was performed between biomarkers of interest on both day 1 and day last separately. All p-values were two-tailed and p-values <0.05 were considered significant.

## Results and discussion

**Fig 1:**
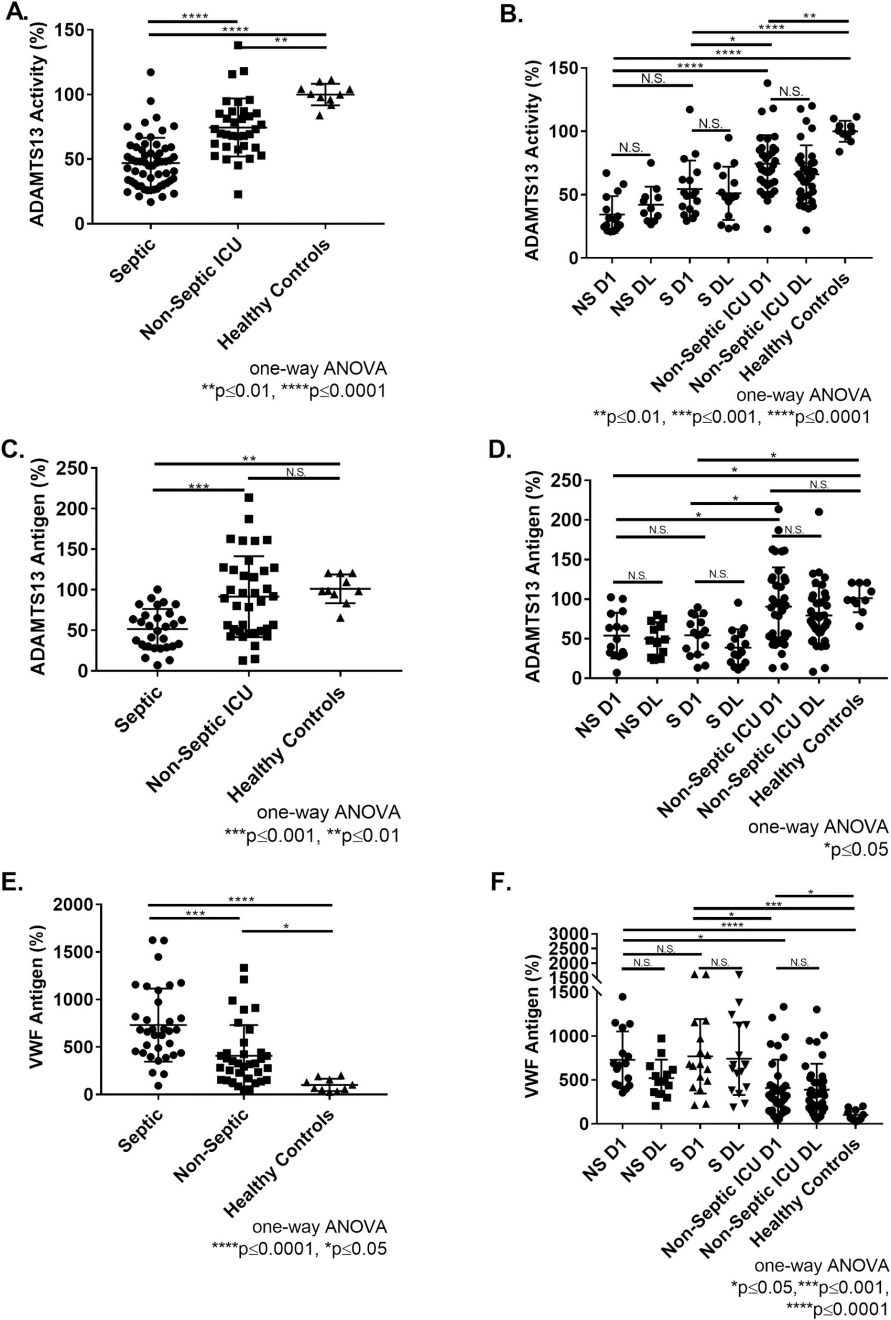
Characterization of ADAMTS13 activity, ADAMTS13 antigen, and VWF antigen in ICU patient samples. **(A)** ADAMTS13 activity in septic patients, non-septic patients, and healthy controls (day 1). **(B)** ADAMTS13 activity in non-survivors septic (NS), survivor septic (S), non-septic, and healthy controls at day 1 and day last. **(C)** ADAMTS13 antigen in septic patients, non-septic patients, and healthy controls (day 1). **(D)** ADAMTS13 antigen in non-survivors septic, survivor septic, non-septic, and healthy controls at day 1 and day last. **(E)** VWF antigen levels in septic patients, non-septic patients, and healthy controls (day 1). **(F)** VWF antigen in non-survivor septic, survivor septic, non-septic, and healthy controls at day 1 and day last. All ADAMTS13 activity, ADAMTS13 antigen, and VWF antigen values normalized to pooled healthy control plasma and expressed as percentage (mean±SD). Dunnett’s multiple comparisons test (one-way ANOVA). Paired t-test performed to assess change between day 1 and day last. ****p≤0.0001, ***p≤0.001, **p≤0.01, *p≤0.05, and N.S. p>0.05.

The baseline characteristics of 40 septic and 40 non-septic ICU patients are summarized in Table 1. Plasma levels of ADAMTS13 activity and antigen were measured in these patients on the first and last days of their ICU stay. Compared to healthy controls, ADAMTS13 activity was reduced in septic patients (47±20%, p≤0.0001) and non-septic patients (75±23%, p≤0.01) (Fig 1A). Moreover, septic patients had reduced ADAMTS13 activity relative to non-septic patients (p≤0.0001) (Fig 1A). Within septic patients, no significant difference was observed between the reduced activity of ADAMTS13 between septic non-survivors (34±15%) and survivors (54±23%) on day 1 (Fig 1B). Significant difference in ADAMTS13 activity was observed for septic non-survivors (34±15%, p≤0.0001) and survivors (54±23%, p≤0.05) compared to non-septic patients (75±23%) on day 1 (Fig 1B). ADAMTS13 activity was significantly reduced among all patient groups compared to healthy controls (100±8%) (Fig 1B). Moreover, ADAMTS13 activity remained reduced as no significant improvement was observed in any patient groups between day 1 and day last (Fig 1B). ADAMTS13 antigen levels were reduced in septic patients (52±25%) relative to healthy controls (p≤0.01) and non-septic patients (p≤0.001) (Fig 1C). Similar to ADAMTS13 activity, no significant difference was observed between the reduced antigen of ADAMTS13 between septic non-survivors (54±29%) and survivors (54±24%) on day 1 (Fig 1D). Significant difference in ADAMTS13 antigen was observed in septic non-survivors (54±29%, p≤0.05) and survivors (54±24%, p≤0.05) compared to non-septic patients (91±50%) on day 1 (Fig 1D). Non-septic ICU patients exhibited normal ADAMTS13 antigen, with large variation from mean on Day 1 (91±50%, p>0.05) and Day Last (79±37%, p>0.05) relative to healthy controls (100±18%) (Fig 1C and 1D). ADAMTS13 antigen remained low as no significant improvement was observed in any patient groups between day 1 and day last (Fig 1D). In sum, on day 1 and day last ADAMTS13 activity and antigen was reduced in sepsis ICU patients and to a lesser extent in non-sepsis ICU patients.

**Table 1:** Patient characteristics. Various patient characteristics recorded upon admission to ICU. Healthy controls were not admitted.

	Non-Survivors Septic	Survivors Septic	Non-Septic ICU	Healthy Controls
**Patient Samples**	20	20	40	10
**Age**	Avg: 67.0±11.7, Min/Max: 41, 84	Avg: 61.5±16.4, Min/Max: 28, 89	Avg: 57.9±17.3, Min/Max: 23, 96	Avg: 46.0±12.3, Min/Max: 27, 65
**Gender (female)**	35%	38%	48%	47%
**Apache II**	Avg: 28.0±6.6, Min/Max: 17, 44	Avg: 22.3±9.6, Min/Max: 12, 51	Avg: 19.1±7.7, Min/Max: 5, 39	
**Day Last (Days)**	10.6±8.8 days	5.7±3.0 days	5.0±2.2 days	
**MODS**	Avg: 10.1±2.7, Min/Max: 7, 14	Avg: 6.1±2.9, Min/Max: 0, 11	Avg: 4.7±3.2, Min/Max: 13, 0	
**SOFA**	Avg: 10.1±2.7, Min/Max: 7, 16	Avg: 7.1±3.1, Min/Max: 1, 14	Avg: 9.0±2.6, Min/Max: 4, 14	
**Mechanical Vent**	100%	93.75%	95%	
**Vasopressors**	65%	37.5%	45%	
**% Medical Intervention**	85%	100%	75%	
**Protein C**	56±37%	72±47%	85±43%	
**Platelets**	168±97%	218±97%		
**Chronic Disease**	90%	18%	50%	
**Liver Disease**	5%	0%	7.5%	
**Diabetes**	30%	44%	25%	
**Cong Heart Failure**	25%	0%	5%	
**Isch Heart Disease**	25%	13%	15%	
**Chr Lung Disease**	40%	19%	10%	
**Cancer**	25%	13%	8%	
**Chr Renal Insuff**	25%	6%	8%	
**Chr Dialysis**	5%	0%	0%	
**Brain Injury**	5%	0%	3%	
**Other Immune**	15%	25%	8%	
**Lung**	50%	31%		
**Blood**	20%	13%		
**Urinary Tract**	15%	6%		
**Other**	5%	50%		
**Gram–ve**	10%	0%		
**Gram +ve**	30%	18%		
**Fungus**	15%	25%		
**Mixed**	20%	6%		

VWF antigen levels were elevated in septic patients (730±384%, p≤0.0001) relative to non-septic patients (463±460%, p≤0.05) and healthy controls (100±65%) (Fig 1E). VWF antigen was elevated in non-septic patients (463±460%, p≤0.05) compared to healthy controls (100±65%), but to a lesser extent (Fig 1E). No significant difference was observed in VWF antigen of septic non-survivors and survivors on Day 1 (Fig 1F). However, VWF antigen of septic non-survivors (729±323%, p≤0.05) and survivors (769±424%, p≤0.05) patients were significantly elevated compared to non-septic patients (Fig 1F). All patient cohorts displayed significantly elevated VWF antigen compared to healthy controls (Fig 1F). In sum, VWF levels were elevated on day 1 and remained elevated on day last in sepsis patients and to a lesser extent in non-sepsis ICU patients.

**Fig 2:**
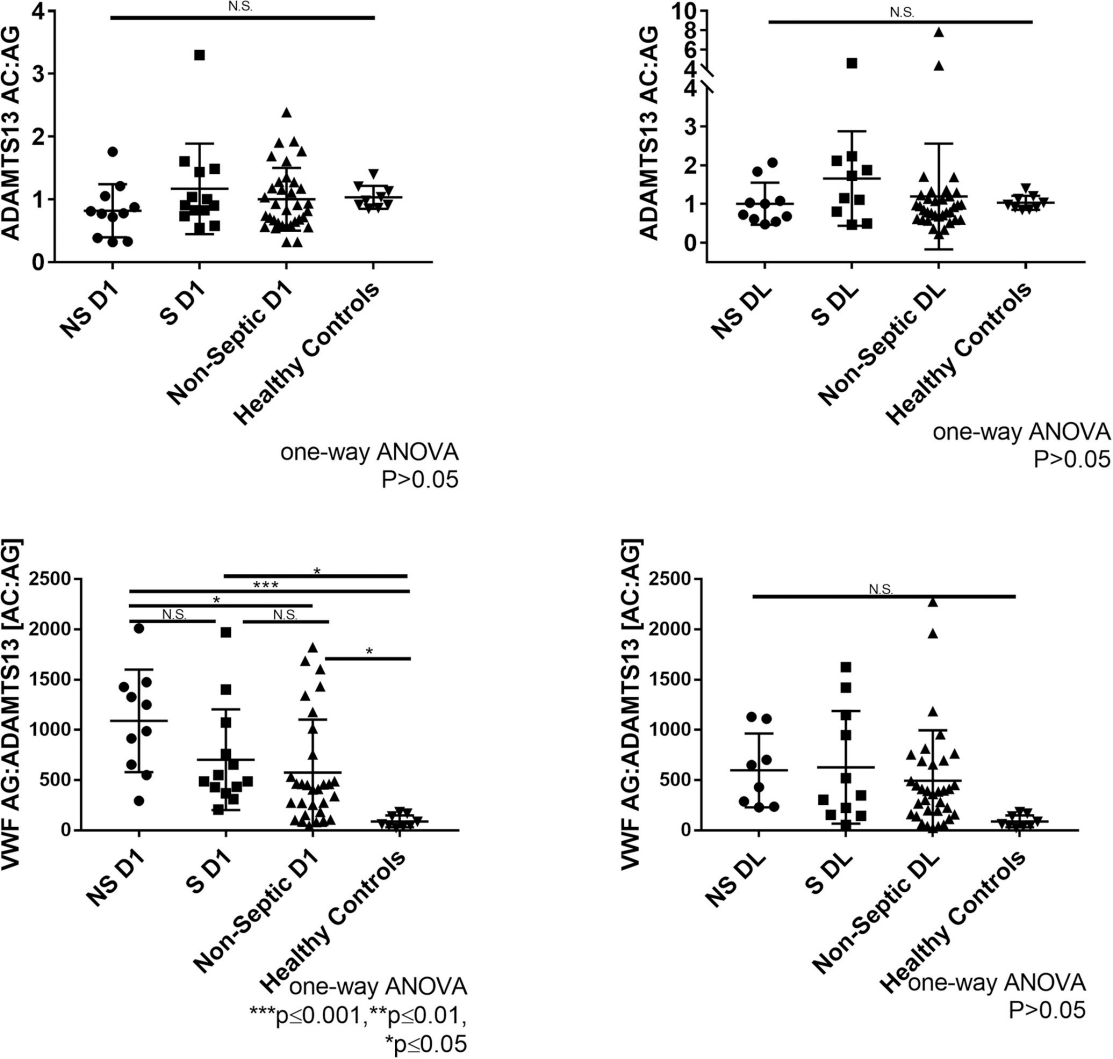
Comparison of ratio of ADAMTS13 activity, ADAMTS13 antigen, and VWF antigen in ICU patient samples. **(A)** Day 1 ADAMTS13 activity to antigen ratio in non-survivor septic (NS), survivor septic (S), non-septic, and healthy controls. **(B)** Day last ADAMTS13 activity to antigen ratio in non-survivor septic, survivor septic, and non-septic ICU. **(C)** Day 1 ratio of VWF AG:ADAMTS13[AC:AG] in non-survivor septic, survivor septic, non-septic, and healthy controls **(D)** Day last ratio of VWF AG:ADAMTS13 [AC:AG] in non-survivor septic, survivor septic, non-septic, and healthy controls. ADAMTS13 activity, ADAMTS13 antigen, and VWF antigen values normalized to pooled healthy control plasma and expressed as a percentage (mean±SD). Dunnett’s multiple comparisons test (one-way ANOVA). ****p≤0.0001, ***p≤0.001, **p≤0.01, *p≤0.05, and N.S. p>0.05.

ADAMTS13 activity and ADAMTS13 antigen levels were correlated on Day 1 (r = 0.3647, p = 0.0014) and at Day Last (r = 0.3377, p = 0.0059). The ratio of ADAMTS13 activity to antigen was similar among all cohorts (p>0.05) (Fig 2A and 2B). This suggests specific activity of ADAMTS13 remains unchanged in septic and non-septic ICU patients. Therefore, reduced ADAMTS13 function in circulation is likely due to a reduction in circulating levels. However, we do acknowledge that with ADAMTS13 assays, activity can be significantly affected by truncated forms of ADAMTS13, autoantibodies, and plasma compounds that may interfere with ADAMTS13 activity but not antigen [20]. A negative correlation was observed between ADAMTS13 activity and VWF antigen (Day 1: r = -0.4283, p = 0.0001; Day Last: r = -0.2728, p = 0.0267). A negative trend was observed between ADAMTS13 antigen and VWF antigen; however the trend was not statistically significant (Day 1: r = -0.1015, p = 0.3797; Day Last: r = -0.2125, p = 0.0774). The lack of significance may be attributed to large variation of ADAMTS13 antigen.

Next, we examined the relationship between VWF[Ag] and ADAMTS13[Ac:Ag] ratio to assess the imbalance between VWF and ADAMTS13. At day 1, VWF[Ag]:ADAMTS13[Ac:Ag] was significantly elevated in both septic (NS: 1090±510, p≤0.001); (S:704±501, p≤0.05) and non-septic ICU patients (576±528, p≤0.05) relative to healthy controls (89±60) (Fig 2C). The imbalance between VWF and ADAMTS13 seems to correspond with severity of sickness. At day last, VWF[Ag]:ADAMTS13[Ac:Ag] remain elevated in both septic (NS: 598±368, p = 0.1193; S: 627±561, p = 0.0578) and non-septic ICU patients (494±502.4, p = 0.1025), but are not significant relative to healthy controls (89±60) due to large variation from mean (Fig 2D). In regards to non-survivor septic patients, the change observed between day 1 and day last in VWF[Ag]:ADAMTS13[Ac:Ag] is due to most patients not surviving past day 1. Together, this data suggests that the imbalance between VWF and ADAMTS13 observed in both septic and non-septic ICU patients contributes to a procoagulant state.

**Fig 3:**
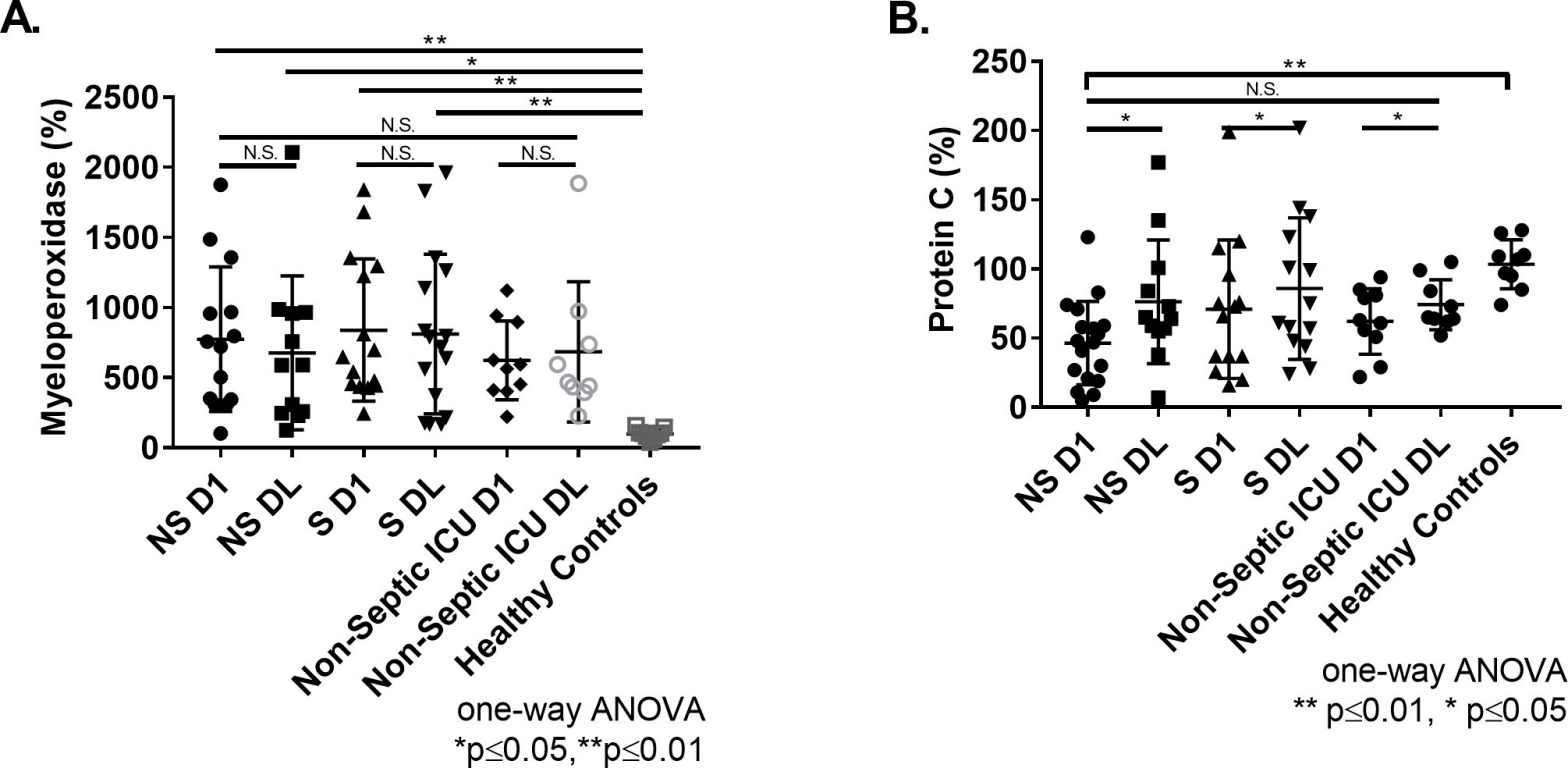
Comparison of Myeloperoxidase and Protein C in ICU patient samples. **(A)** MPO levels in non-survivor septic (NS), survivor septic (S), non-septic, and healthy controls at day 1 and day last. **(B)** Protein C levels in non-survivor septic, survivor septic, non-septic, and healthy controls at day 1 and day last. MPO and Protein C values normalized to pooled healthy control plasma and expressed as a percentage (mean±SD). Dunnett’s multiple comparisons test (one-way ANOVA). Paired t-test performed to assess change between Day 1 and Day Last. ****p≤0.0001, ***p≤0.001, **p≤0.01, *p≤0.05, and N.S. p>0.05.

Extensive neutrophil degranulation occurs during sepsis, and has been shown to inhibit proteolytic cleavage of VWF by ADAMTS13 [21]. Therefore, myeloperoxidase (MPO) levels were measured as a marker of neutrophil degranulation. MPO was elevated in septic non-survivors on day 1 (774±516%, p≤0.01) and day last (677±550%, p≤0.05), and in survivors on day 1 (839±507%, p≤0.01) and at day last (812±569%, p≤0.01) compared to healthy controls (100±36%) (Fig 3A). No difference was observed between septic and non-septic patients (Fig 3A). In non-septic patients, MPO were elevated, but not significantly different from healthy controls on day 1 (624±284%, p = 0.09) and at day last (684±500%, p = 0.06). (Fig 3A). Further, MPO levels did not correlate with ADAMTS13 activity on either day 1 (r = 0.0003, p = 0.9243) or at day last (r = 0.0278, p = 0.7686) or with ADAMTS13 antigen on either day 1 (r = 0.0494, p = 0.7686) or at day last (r = -0.0035, p = 0.9833).

Protein C is a natural anticoagulant that is known to be consumed during sepsis due to ongoing systemic activation of coagulation [22]. Protein C antigen was decreased in non-survivor day 1 patients (46±30%, p≤0.01) (Fig 3B). No difference in Protein C levels was observed between any other patient groups at day 1 and day last (Fig 3B). Protein C levels did not correlate with and ADAMTS13 activity on day 1 (r = 0.3035, p = 0.0921) and day last (r = 0.0865, p = 0.1365) or with ADAMTS13 antigen on day 1 (r = 0.0332, p = 0.9040) and day last (r = 0.0042, p>0.7039). Consistent with previous studies in septic patients [23], Protein C levels recovered in patients throughout their hospital stay. Across all patient cohorts, Protein C levels increased between day 1 and day last (p≤0.05) (Fig 3B). By comparison no change was observed in ADAMTS13 activity, ADAMTS13 antigen, VWF antigen, or MPO between day 1 and day last among all patient groups. Therefore, dysregulation between ADAMTS13 and VWF may extend beyond the ICU in sepsis survivors. Deidentified raw data is provided in S1 Data.

The cause of ADAMTS13 reduction in sepsis patients remains unknown. Activated coagulation proteases can be generated in high concentrations in sepsis patients experiencing DIC. Many of these proteases, including thrombin and plasmin, have been shown to degrade ADAMTS13 and reduce its capacity to regulate VWF [12, 13]. Whether these proteases contribute to reduced ADAMTS13 concentration in sepsis patients is currently not known, and worth future investigation. Recent reports suggest that human neutrophils (HNPs) mimicking the VWF A2 domain may serve to inhibit ADAMTS13 activity [21]. We found no evidence that activated neutrophils in sepsis patients release sufficient levels of HNPs to reduce ADAMTS13 activity beyond what would be expected based on antigen levels. Therefore, we conclude that reduction in ADAMTS13 antigen is the primary mechanism of reduced ADAMTS13 function in sepsis patients. However, we are careful to not exclude the possibility that locally high concentration of HNPs and/or activated coagulation proteases may play a temporally or spatially constrained role in regulating ADAMTS13 activity.

It is possible that increased levels of VWF can bind and clear ADAMTS13 from circulation due to differences in their circulating half-lives [2, 4]. Consistent with this hypothesis, cecal ligation and puncture (CLP)-induced sepsis resulted in an increase in VWF secretion and a decrease in ADAMTS13 [14]. In contrast, ADAMTS13 remained normal in *Vwf*-/- mice subjected to CLP surgery. These studies suggest that VWF is a major determinant of decreased ADAMTS13 activity in the CLP model of polymicrobial abdominal sepsis. From a mechanistic point of view, massive release of VWF from the endothelium and subsequent reductions in ADAMTS13 may result in persistently high molecular weight VWF [24]. Such high molecular weight VWF is known to contribute to microvascular thrombosis through enhanced reactivity to platelets [24]. Future studies will examine how changes to ADAMTS13 and VWF affect multimer distribution, and whether this contributes to the thrombosis risk in sepsis patients. Moreover, potential confounders may not have been taken into account as septic patients seem to show higher number of chronic diseases and underlying factors compared non-septic patients.

There are potential consequences of reduced ADAMTS13 activity and elevated VWF levels. ADAMTS13 and VWF levels did not normalize in survivors at discharge, unlike Protein C which was previously shown to normalize in septic patients at ICU discharge [23]. The prolonged imbalance in VWF and ADAMTS13 may contribute to microvascular thrombosis, ischemic stroke, and organ damage observed in some patients who survive sepsis [25]. Sepsis survivors are also at a higher risk for cognitive impairment and cardiovascular disease [26]. However, the correlation between ADAMTS13 or VWF with these outcomes in sepsis survivors is currently not known. In addition, the time to normalization of ADAMTS13 and VWF in sepsis survivors post ICU discharge remains unknown. This study demonstrates that ADAMTS13 and VWF are dysregulated in sepsis patients and to a lesser extent in non-septic patients, and that these parameters do not normalize in survivors at discharge from the ICU.

## Supporting information

S1 Data Deidentified raw patient data. Raw Patient values for ADAMTS13 activity, ADAMTS13 antigen, VWF antigen, and MPO at day 1 and day last.
(XLSX)
